# Alimentary Infections by Tick-Borne Encephalitis Virus

**DOI:** 10.3390/v14010056

**Published:** 2021-12-30

**Authors:** Martina Ličková, Sabína Fumačová Havlíková, Monika Sláviková, Boris Klempa

**Affiliations:** Biomedical Research Center, Institute of Virology, Slovak Academy of Sciences, 84505 Bratislava, Slovakia; martina.lickova@savba.sk (M.L.); virusaha@savba.sk (S.F.H.); monika.slavikova@savba.sk (M.S.)

**Keywords:** tick-borne encephalitis virus, TBEV, goats, sheep, cows, milk, alimentary infection

## Abstract

Tick-borne encephalitis virus (TBEV) causes serious the neurological disease, tick-borne encephalitis (TBE). TBEV can be transmitted to humans by ticks as well as by the alimentary route, which is mediated through the consumption of raw milk products from infected ruminants such as sheep, goats, and cows. The alimentary route of TBEV was recognized in the early 1950s and many important experimental studies were performed shortly thereafter. Nowadays, alimentary TBEV infections are recognized as a relevant factor contributing to the overall increase in TBE incidences in Europe. This review aims to summarize the history and current extent of alimentary TBEV infections across Europe, to analyze experimental data on virus secretion in milk, and to review possible alimentary infection preventive measures.

## 1. Introduction

Tick-borne encephalitis virus (TBEV) is an increasingly important human pathogen causing tick-borne encephalitis (TBE), one of the most significant neurological zoonotic diseases in humans. The consumption of raw milk obtained from a variety of animal species, such as cows, goats, and sheep, plays a considerable role in the recent increase of tick-borne encephalitis virus cases across Europe. Despite the fact that this alimentary route of TBEV infection has been known for decades, it is often neglected in the scientific literature. Shortly after the alimentary route of TBEV infection was first recognized, numerous experimental studies involving domestic ruminants were performed in Eastern and Central European countries, such as the former Czechoslovakia and Soviet Union, in the 1950s and 1960s. These studies are nowadays difficult to find due to the limited availability of the original publications. One of the scopes of this review is to summarize and preserve the findings of those pioneering experimental animal studies focused on domestic ruminants performed in the early days of TBEV research. This review also summarizes the current extent of alimentary infections across Europe and reviews possible alimentary infection preventive measures.

## 2. Tick-Borne Encephalitis Virus (TBEV)

Tick-borne encephalitis (TBE) is one of the most important tick-borne viral diseases in Europe and Asia. Worldwide, more than 10,000 cases are reported annually [1]. The causative agent is tick-borne encephalitis virus (TBEV), which belongs to the genus *Flavivirus*, of the family *Flaviviridae*, which is mainly distributed in Europe and Asia [2,3]. TBEV has an 11 kb long positive single-strand RNA genome encoding a single polyprotein that is cleaved into three structural proteins (C, M, E) and seven non-structural proteins (NS1, NS2A, NS2B, NS3, NS4A, NS4B, NS5) [4,5].

Phylogenetic analysis has revealed three main subtypes of TBEV: European (TBEV-Eu), Siberian (TBEV-Sib), and Far Eastern (TBEV-FE) [6,7]. Recently, two additional subtypes were proposed, the Himalayan (TBEV-Him) [8] and Baikalian (TBEV-Bkl) TBEV subtypes. TBEV-Him was identified in the wild rodent *Marmota himalayana* in the Qinghai-Tibet Plateau in China [8], while TBEV-Bkl was found in East Siberia near Lake Baikal and in Northern Mongolia [9,10]. Furthermore, classification based only on genomic data defining seven TBEV subtypes, TBEV-Eu, TBEV-Sib, TBEV-FE, TBEV-2871 (TBEV-Ob), TBEV-Him, TBEV-178-79 (TBEV-Bkl1), and TBEV-886-84 (TBEV-Bkl-2), was recently proposed by Deviatkin et al. [11].

The TBEV-Eu subtype is prevalent across Europe, including the European part of Russia, whereas the TBEV-Sib and TBEV-FE subtypes are present mainly in Asia. In some areas, two or all three main subtypes coexist (e.g., in the Baltic States, Siberia, and Ukraine) [12].

The clinical course of TBEV infections can range from asymptomatic infections and mild febrile disease with the complete recovery of patients to severe or even fatal encephalitis. TBE manifests in flu-like symptoms such as fever, headache, nausea, ataxia, tremor, paresis, paralysis, fatigue, in some cases vomiting, and even death. It can result in a variety of neurological manifestations including meningoencephalitis. The incubation period in most cases is between 7–10 days (with a reported minimum and maximum being 4 and 28 days, respectively) after infection by an infected tick bite. Infection through unpasteurized milk and dairy products usually results in a shorter incubation period of approximately 3–4 days [4].

The severity of the clinical outcome depends on the viral subtype. TBEV-FE-associated human infections are usually the most severe, while the TBEV-Sib is milder but results in chronic disease more often. TBEV-EU-associated cases are usually milder, without serious sequelae, and have a typical biphasic course with fever during the first phase and neurological disorders of differing severity during the second phase. TBEV-Eu is associated with a case fatality rate (CFR) of 0.5–2%, TBEV-Sib with a 2–3% CFR, and TBEV-FE with a CFR of up to 40% [4,13,14,15].

## 3. Routes of TBEV Transmission

**Figure 1:**
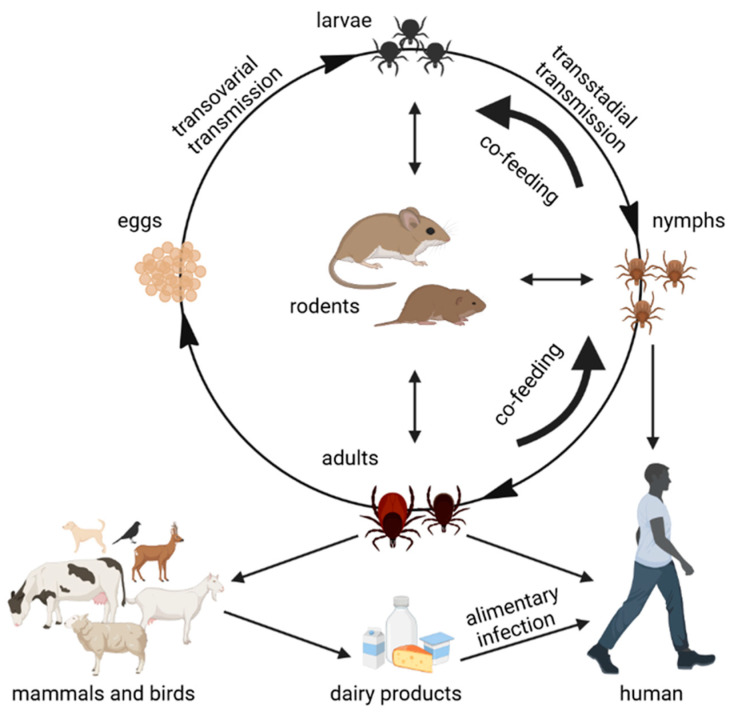
Routes of TBEV transmission within the life cycle of ixodid ticks. The *Ixodes ricinus*, *I. scapularis* and *Dermacentor reticulatus* ticks are able to maintain TBEV in nature. The life cycle of ticks consists of four developmental stages (larva, nymph, adult, and egg). Each parasitic stage (except egg) needs to take a blood meal on a suitable host to develop into the next stage. The main animal reservoirs for TBEV are rodents. Larger mammals and birds may act as hosts for adult ticks. TBEV infects ticks while they are feeding on an infected reservoir. The main route of TBEV maintenance in nature is non-viraemic transmission from nymphs to naive larvae while co-feeding on the same rodent host. TBEV can be transmitted to humans via a tick bite (mostly nymphs) and via alimentary infection through the consumption of raw milk products from TBEV-infected ruminants (goats, sheep, and cattle) (Figure was created by BioRender).

In nature, TBEV is circulating in small, geographically defined areas often termed as “natural foci”. It is maintained in a cycle involving ticks as vectors and small rodents, insectivores, and large mammals (wild animals, domestic grazing ruminants, birds) as hosts [16,17] (Figure 1). The principal tick vectors of TBEV are *Ixodes ricinus*, which is associated with TBEV-Eu, and *I. persulcatus*, which is associated with Siberian and Far Eastern subtypes [12]. Ticks can get infected during feeding on viraemic hosts or by co-feeding with the infected tick [18,19]. Moreover, the virus can also be vertically transmitted from an infected adult female tick to its offspring through transovarial transmission [20,21]; however, this seems to be ineffective and not decisive for the virus maintenance cycle. Virus transmission between co-feeding ticks, termed non-viraemic transmission (NVT) and typically occurring between tick nymphs and larvae, is considered to play a crucial role in the TBEV transmission cycle (reviewed in [22,23]). Consequently, the intimate ecological associations of *I. ricinus* and *I. persulcatus* ticks, with their vertebrate hosts leading to the co-feeding of larvae and nymphs on the same hosts, might explain why these two-tick species are recognized as the main vector ticks of TBEV, despite evidence that at least 22 tick species have been associated with TBEV [24,25]. Nevertheless, recent experimental findings, together with several observational field studies, indicate that *Dermacentor reticulatus* might be an additional underrecognized, but biologically effective, TBEV vector [26,27].

Besides rodents being the most important TBEV hosts, large mammals such as cows, goats, sheep, and many other wild and domestic animals (including dogs) can be infected and become viraemic as well [28]. Among domestic animals, goats are most commonly infected with TBEV due to their way of grazing (preferring brushes rather than grass). 

Humans are accidental hosts of TBEV. They can be infected through tick bites from all tick stages as the main route of transmission or by alimentary infection through the consumption of raw milk and milk products from infected grazing ruminants [29]. 

Occasional reports of additional TBEV transmission routes have been described, including aerosol infections among laboratory personnel [30,31], blood transfusion [32], organ transplantation [33], the slaughtering of a viraemic goat [34], and transmission from a viraemic mother to her baby via breast milk [35].

## 4. Brief History of the First Recognized Alimentary Infections

Alimentary-transmitted TBE, historically called biphasic milk fever, was described for the first time in the European part of Russia between 1947 and 1951. This form of disease was associated with the consumption of milk from goats and typically affected groups of people who consumed unpasteurized milk from the same source (e.g., families). However, the etiologic agent of the diseases was not known at that time [36]. Later, TBEV-infected goats developing subclinical infections were identified as the source of infection for people consuming their milk [37,38].

In Slovakia (as a part of the former Czechoslovakia), the consumption of raw milk products was recognized for the first time as a previously unknown mode of TBEV transmission because of a well described 1951 outbreak in the city of Rožňava (Southern Slovakia) with at least 660 people infected, of which 271 were hospitalized with meningoencephalitis. Most of the patients consumed inadequately heat-treated cow’s milk mixed with goat’s milk distributed from a local dairy. An epidemiological investigation revealed that the TBEV infection might have been not only due to tick bites but also the consumption of raw goat’s milk [39]. In the following years, several local epidemics of alimentary infections were observed in western and central Slovakia, and in many cases, whole families became infected after the consumption of raw goat’s milk. During 1952–1953, alimentary TBE cases represented 23.9% of all reported TBEV infections [39,40,41].

Initially, only goats and their milk were suspected to be the source of alimentary TBEV infections. Gradually, evidence that sheep and cows might play similar role in infections accumulated as well. Blaškovič [39] reported two cases of TBE in children through the consumption of raw cow’s milk. A small epidemic associated with the consumption of fresh, raw sheep cheese and whey was reported in 1974 [40]. The epidemic in Rožňava provided the impetus for TBEV research in Slovakia and led to a series of experimental studies involving domestic animals. These experimental TBEV infections revealed that not only goats but also sheep and cows became seroconverted, and infectious virus was detected in their milk [42,43,44,45,46]. The growth of TBEV in sheep’s milk was confirmed experimentally [43,45].

Outbreaks of TBE after the consumption of raw goat’s milk were also repeatedly reported in the former Soviet Union. Considerably high proportions of alimentary infections among all TBE cases were reported in the north-western part of the country (59.5%; [47]), Perm region (4.2%; [48]), Sverdlovsk region (48%; [49]), and Udmurstia (26.7%; [50]). In the Czech Republic, alimentary infections were reported in Hradec Králové [51,52] and in 10% of TBE patients in the Ostrava area [53]. Tongeren et al. [54] reported alimentary infections in 18% of TBE cases in Austria.

## 5. Alimentary Infection in Europe 

Since 2012, TBE has been listed as a notifiable disease in the European Union [55], and TBEV is endemic in 27 European countries (Stokholm European Centre for Disease Prevention and Control, 2012, www.ecdc.europa.eu (accessed on 23 December 2021)). Approximately 1% of all TBEV infections in humans are contracted by the alimentary route [3,12,13]. This route of transmission typically leads to local outbreaks, which are mostly reported in Eastern and Central Europe; however, small outbreaks of foodborne TBE have also been reported in Western Europe [40,56,57,58,59]. 

**Figure 2:**
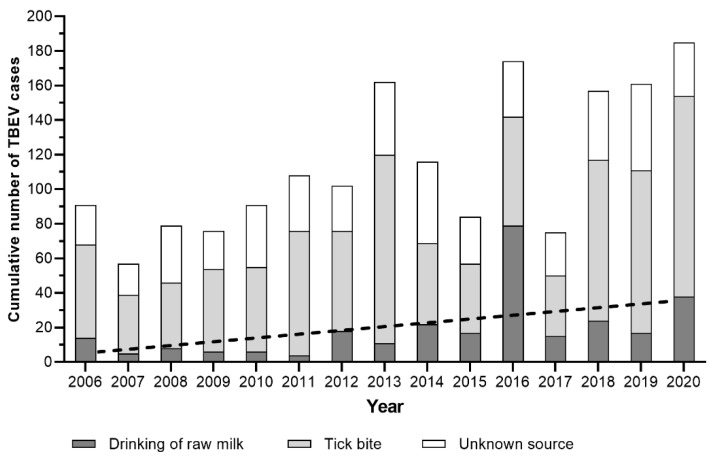
Epidemiology anamnesis of human cases of TBEV in Slovakia (source: www.uvzsr.sk (accessed on 23 December 2021)). The dashed line represents a linear trendline for the alimentary infections.

In Slovakia, 10 alimentary outbreaks were reported during the years 1951–2000, with the number of cases varying between 3 and 271. The largest outbreak was reported in the city of Rožňava in 1951 [39], while the majority of the outbreaks occurred in May [60]. Kerlik et al. [61] reported 26 alimentary outbreaks of TBE in the years 2007–2016 and during that time, 17% of all TBEV infections were through raw milk consumption. According to data from the Slovak Institute of Public Health (www.uvzsr.sk (accessed on 23 December 2021)), this trend has been continuing, as illustrated in the period from 2017–2020, in which alimentary infections represented 16.3% of all recognized TBE cases (Figure 2).

Croatia has a low incidence of TBE (1/100,000) [62], and sporadic TBE cases are mostly associated with tick bites. However, the first cluster of TBE cases linked to the consumption of raw goat’s milk and cheese was reported in 2015. The outbreak was confined to two families and most patients had mild clinical features without neurologic sequelae [63]. Ilic et al. [64] reported another outbreak of TBEV in 2019 linked to consumption of raw goat’s milk, where three of the six patients had neurological involvement.

Balogh et al. [65] reported a TBE outbreak in Lakhegy, Hungary in August 2007. During this outbreak, 31 of the 154 exposed persons became ill. All the patients drank raw goat’s milk and none of them had a tick bite. Twenty-five patients had typical symptoms of biphasic TBE with neurological symptoms during the second phase. Six patients had non-specific symptoms. TBE infection in the 25 patients with neurological symptoms was confirmed by serological tests, in contrast to the patients without the second phase of illness [65]. Caini et al. [58] reported alimentary infections due to drinking unpasteurized cow’s milk in Hungary in 2011, where 11 out of 103 exposed persons contracted TBE. Overall, during 1953–2011, 4.4% of TBE cases in Hungary were associated with the alimentary route of infection [66].

In 1999, in the north-east Moravia region of the Czech Republic, 21 human cases of alimentary TBE were reported [67], and the source of infection was home-made sheep cheese [67]. In a retrospective survey of pediatric TBE cases between 1960 and 2007, Pazdiora and colleagues [68] confirmed 410 TBE cases in children in West Bohemia, and the consumption of unpasteurized milk was determined in 11 instances. Overall, during 1997–2008, 64 cases of alimentary TBE (0.9% of all human TBEV infections) were reported in the Czech Republic. Children had a 2.5-fold higher risk of alimentary infection in comparison to adults. It would appear that the belief of parents that children consuming unpasteurized dairy were healthier in fact increased risk in children [69].

One of the countries with the highest reported TBE incidence in Europe (8.1–18.6 cases/100,000 population) is Slovenia [70]. Hudopisk et al. [59] reported a small outbreak of TBE among consumers of raw goat’s milk in 2012. Febrile illness occurred in three of four persons 2–3 days after the consumption of milk. The fourth person was vaccinated and remained healthy. TBEV RNA was directly detected in the serum and milk of the suspected goat.

Norway is a country with a low incidence of human TBE. The human cases occur especially in southern Norway (Norwegian Institute of Public Health, 2018). Paulsen et al. [71] found TBEV RNA in unpasteurized milk in 5.4% of the tested animals. They also detected antibodies to TBEV in Arendal with an 88.2% positivity rate in animals.

In the pre-vaccination era, Austria had the highest recorded morbidity of TBE in Europe, although this could have been a consequence of its advanced early diagnostic tools. Nevertheless, in regions with abundant TBEV foci, TBE accounted for more than 50% of all viral meningoencephalitides. The decline was most dramatic in the province of Carinthia, which had the highest morbidity rate in the pre-vaccination era. During 1973–1982, a total of 1550 cases of TBE were recorded, compared with 17 cases in the years 2000–2003 [72]. Alimentary cases were also documented. Holzmann et al. [57] reported a TBE outbreak comprising six cases in a mountain region in western Austria in July 2008. In this incident, seven persons ate self-made cheese prepared from a mixture of non-pasteurized goat’s and cow’s milk: four people developed typical biphasic TBE, two were clinically asymptomatic, and one person remained uninfected because of a gastric banding (he vomited shortly after eating the cheese) [57]. Interestingly, the goats were grazing at 1500 m above sea level, supporting the trend of *I. ricinus* ticks spreading to higher altitudes [73,74,75]. Most recently, four cases of alimentary TBE (two clusters) were reported in 2020 [76].

Milk-borne TBE epidemics were also reported in Poland in the Olsztyn Voivodship in 1974, the Kielce Voivodship in 1995, and in the Wroclaw Voivodship in 1996. The source of the infection was unpasteurized cow’s milk [77] and goat’s milk [78,79]. Król et al. [80] documented a small outbreak in the Podlaskie Voivodship in four monks who developed mild biphasic TBE following the consumption of raw goat’s milk.

In Estonia, 27 cases of TBEV were reported in 2005. All cases were associated with the consumption of raw goat’s milk that had been offered to customers to taste at a supermarket in Tallinn in May 2005 as part of a promotion [81]. Other recent alimentary outbreaks of TBEV notified in Estonia include a family outbreak involving three people in 1990, an outbreak involving ten military recruits in 1992, and a household outbreak involving three family members and one guest in 2004 [82].

In Germany, alimentary TBEV infections are not regarded as epidemiologically important. A food-borne outbreak of TBEV was reported in 2016 when two TBE cases occurred after the consumption of raw goat cheese. The outbreak was rather unusual as none of the additional 30 consumers of the same cheese presented any neurological symptoms [83]. The index of manifestations (exposed vs. infected persons) during milk-borne outbreaks is usually much higher [69,84]. Important observations were made during an alimentary outbreak which occurred in 2017. Epidemiological investigation of a single hospitalized TBE patient revealed an alimentary, goat’s milk-associated outbreak with 27 exposed persons. Of the 20 people with available medical information, 13 were infected and reported symptomatic disease. Of the six vaccinated exposed persons, only one person, who was vaccinated more than 15 years ago, developed disease. In contrast, 12 out of the 14 unvaccinated exposed persons developed TBE. Thus, this outbreak showed that vaccination also protects against the alimentary transmission of TBEV [85]. 

No alimentary infections have been reported in Lithuania. However, observed TBEV seropositivity in humans has correlated with cases of meningoencephalitis and with the consumption of raw goat’s milk, but this is without laboratory confirmation [86], suggesting supposed alimentary transmission routes.

Alimentary TBE cases have also been reported also in Romania. During 1999–2006, 17 cases of alimentary infections out of 37 TBE cases total were documented [87,88].

Altogether, alimentary TBEV infections were reported in at least 10 European countries, but their extent across countries varies. The countries with the highest reported numbers of TBEV alimentary cases are the Czech Republic [69], Poland [78], Hungary [58], and Slovakia [61] (Table 1). Some countries have reported only several outbreaks (Croatia, Germany, and Slovenia; Table 1). It would appear that the countries with the highest number of alimentary TBEV infections are those with a more agricultural structure. However, recent cases in Germany and Austria show that alimentary outbreaks can also happen in countries with highly industrialized agriculture. Consequently, we have to consider milk-borne TBE as a potential human health risk in the whole TBEV endemic area in Europe. 

## 6. Excretion of TBEV in Milk

Recently, the number of TBE cases that are caused by the consumption of raw milk is increasing. Milk is often consumed raw among farming families and others due to its better taste, simple preparation, higher biological values, or in order to prevent and treat certain diseases [92,93]. However, most of the beneficial nutrients in milk are heat stable and/or largely unaffected by pasteurization. Raw milk, cheese, or dairy products can be contaminated with many pathogens including TBEV [94].

Alimentary transmission of TBEV occurs from viraemic domesticated animals after the consumption of non-pasteurized goat’s milk and cheese [57,58,65] and also from raw sheep and cow’s milk [40,58] in endemic areas. The course of TBEV infection in animals is not very well understood. Domesticated ruminants such as goats, sheep, and cows do not show clinical signs of disease but excrete the virus through their milk. Similarly, antibodies against TBEV have been observed in wild-living ruminants such as roe deer and carnivores such as red foxes without specific neurological symptoms [44,65,95,96]. However, symptomatic infections have occurred in dogs, horses, monkeys [97,98,99], and, in rare instances, in ruminants as well [100].

Ruminants might serve as important sentinel animals for TBEV occurrence in nature and show a persistence of specific antibodies for up to 28 months post infection [101]. Several studies have therefore focused on domestic ruminants as sentinels and have mainly focused on goats and sheep. Goats can be repeatedly infected and excrete TBEV in milk over a long period [38]. TBEV can be isolated from the milk of goats for 3–25 days following infection, is infectious in various milk products such as yoghurt, cheese, or butter, and can be transmitted from goat to kid via milk [94,102]. 

**Figure 3:**
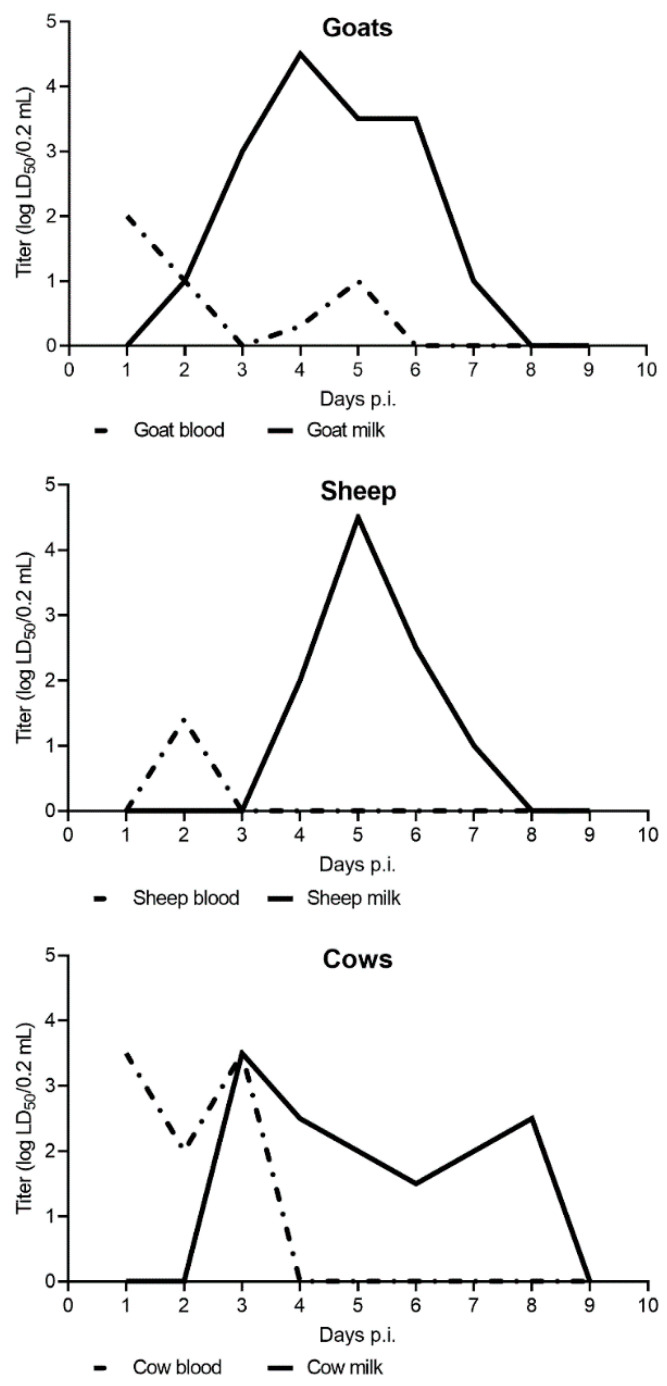
Comparison of TBEV viral titers in milk (full line) and blood (dashed line) of experimentally infected sheep, cows, and goats (adapted from Ref. [84]).

The first experimentally verified excretion of TBEV in the milk and blood of TBEV inoculated goats was reported by Grešíková in 1957 [42] (Figure 3). The highest concentration of virus in the blood was at 24 h post infection (p.i.). After 24 h p.i., the concentration of TBEV in the blood decreased each day. In milk, the viral load increased from 48 h p.i. until the 4th day p.i., when it reached the maximum. Starting from the 5th and 6th days p.i., the viral load decreased and reached the minimum at day 7 p.i. (Figure 3). The shedding of TBEV via goat’s milk was also confirmed after the peroral infection of mice. All mice infected via the goat’s milk had died. Half of the infected goats had inapparent infections indicating that they might serve as long-lasting reservoirs of TBEV [42].

Benda [103] confirmed the excretion/viraemia of TBEV via goat’s milk and showed the virus had higher titers and persisted longer in milk than in blood. The excretion of TBEV through goat’s milk was also confirmed in other experimental studies [104].

Consistent with goats, studies of infected sheep revealed that TBEV is detected from 1–6 days in blood and 2–7 days in milk, with peak viral loads occurring on day 2 in blood and day 5 in milk, with milk having higher and more persistent viral loads overall relative to blood [43]. Excretion of TBEV via sheep’s milk has also been confirmed after subcutaneous infection of the virus as well as after the feeding of infectious *I. ricinus* ticks on animals [43,45].

TBEV was also isolated from the milk of infected cows. However, the viral loads did not reach levels as high as those detected in sheep and goats [44] (Figure 3). Cows inoculated with TBEV showed no clinical signs of disease. The virus was isolated on day 1–5 p.i. from the blood and on day 2–6 p.i. from the milk, both showing variation in individual animals. In general, TBEV titers were considerably higher in milk than in blood during viraemia (Figure 3) [84]. 

## 7. Stability and Survival of TBEV in Milk and Dairy Products

In general, flaviviruses are rapidly inactivated at 50 °C, with 50% of infectivity lost in 10 min and total inactivation of the virus occuring within 30 min at 56 °C [4]. TBEV is an enveloped RNA virus that is relatively sensitive to temperature and detergents. However, TBEV remains infectious in gastric juice for up to 2 h when mixed with milk [105]. TBEV is destroyed in milk only after heating for 30 min at 65 °C. The virus is not destroyed in milk products when the temperature during the preparation is below that value [84]. Balogh et al. [94] demonstrated that this temperature (65 °C, 30 min) is not efficient for complete inactivation of TBEV. Goat’s milk samples with lower/higher content of the virus were tested after heat treatment with three chosen temperatures: room temperature, to simulate the production of cheese and cottage cheese; 65 °C, to simulate heating; and 100 °C, to simulate boiling conditions. Only treatment at 100 °C for 3 min eliminated the infectivity of both pools [94]. 

The stability/infection of TBEV in raw goat’s milk and in a laboratory cell culture medium was studied by Offerdahl et al. [106]. Langat virus (LGTV) was used as a model virus because of its convenience for use as a biosafety level 2 virus as a model for more pathogenic viruses [107]. The virus was added to the fresh, unpasteurized goat’s milk or to the cell culture medium. The milk was incubated for 0, 8, 24, 48, and 72 h at 4 °C or at 22 °C. When the medium was incubated at 4 °C, the titer decreased over the 72 h. Virus incubated in milk was stable at 4 °C for 8 and 24 h. The obtained results demonstrated that LGTV is stable for several days at refrigerated conditions. At 22 °C, viral infectivity modestly decreased within 72 h in the cell culture medium. When incubated in goat’s milk at room temperature, the infectivity decreased within 24 h and was undetectable after 48 h. Moreover, the study also simulated conditions during the pasteurization of milk, involving high temperatures over a short period of time. Milk was heated to 72 °C for 15 s and then immediately cooled to 4 °C. This procedure led to the complete inactivation of the virus. The experimental fermentation of cheese was done at temperatures up to 30 °C following gradual cooling to 22 °C and incubation for 16 h. This procedure reduced the virus in goat’s milk. The virus cultivated in the medium was not totally inactivated. The partial simulation of cheese fermentation reduced the quantity of virus, but the virus was still detectable as a residual. The infectivity of the virus also depended on storage conditions. While the virus was stable for at least 72 h in refrigerated milk, infectious virus was undetectable within 48 h at ambient temperature [106].

Since 1943, both pasteurization conditions involving either a short pasteurization time (72 °C for 15 s) or a soft, holding method (63 °C for 30 min) were confirmed as adequate to destroy a wide range of pathogenic bacteria in milk [108]. These conditions also destroy most of the yeast, molds, in addition to spoilage, pathogenic, and heat-resistant organisms. However, the holding method (63 °C for 30 min) only partially eliminates infectious virus, while the short pasteurization method (72 °C for 10 s) completely inactivated the virus. The short boiling method (100 °C for 3 min) is the safest method to completely destroy infective virus particles [94].

Rónai and Egyed [109] compared the survival of TBEV in pasteurized/unpasteurized goat’s milk and salted/unsalted and spiced/unspiced cheese inoculated with low/high titer of the virus. Both pasteurization methods—soft holding at 63 °C, 30 min, and fast holding at 72 °C, 15 s destroyed virus particles. A low amount of virus was detected for 5–10 days in milk and in unsalted cheese. With higher doses of virus, TBEV was detectable for longer periods in milk (20–25 days) and unsalted cheese (10–15 days), independently of the use of spices. The virus survived in raw milk for 3 weeks at 4 °C and in cheese made from raw milk for 2 weeks. Both pasteurization and salt treatment made goat’s milk and cheese safe for consumption. These findings underline that the safest and simplest way to avoid milk-borne TBE is to boil/pasteurize the milk before drinking it, and if the consumer insists on raw milk, it is important to immunize the goats against TBEV in endemic areas.

## 8. Differences in Clinical Course of Tick- vs. Milk-Borne TBE

There are five main differences between TBE associated with the consumption of raw milk (goat’s, sheep) and tick bites [4].

- Incubation period. After a tick bite, the incubation period is on average 7–14 days (range 4–28 days) [4]. The incubation period after exposure by the alimentary route can be shorter, usually 3–4 days [110] and even as short as two 2 days [59].
- The biphasic form of TBE is more common in milk-borne cases, whereas the biphasic form after a tick bite constitutes only 20–30% of all cases [71].
- Disease severity. Non-severe meningoencephalitis during the second phase of milk fever is more often observed, whereas clinical manifestations of tick-associated TBE are usually more severe, including meningitis, meningoencephalitis, or meningoencephaloradiculitis [4,111].
- Recovery. Patients overcoming milk-borne infections have a high probability of recovering without neurological sequelae (almost 100%), whereas tick-associated TBE is more often associated with long-term disability and death [4].
- Occurrence. TBE after a tick-bite usually occurs in the form of sporadic cases, while alimentary infections are mostly characterized by family incidence or small outbreaks associated with the consumption of raw milk products [59].

It can be concluded that both forms of TBE (tick- and milk-borne TBEV infections) are caused by the same etiologic agents. Biphasic milk fever is considered an epidemiological form of TBE rather than a disease caused by some specific strains of TBEV. The differences in the clinical courses can be explained by differences in the immunological response depending on the route of infection [4].

## 9. Gut Infection

TBEV is relatively sensitive to temperature and detergents but remains infectious in gastric juice (pH 1.49–1.80) for up to two hours [49]. Milk and milk products are quickly moved from the stomach (the first consumed milk reaches the duodenum within minutes) and hydrolytic acid is secreted in the stomach between 45–60 min after the consumption of the milk. Therefore, it has been suggested that the human digestive tract is an efficient route of infection. This has been confirmed by the experimental infection of mice, which became infected with TBEV after feeding [105]. The pathogen is transmitted by the intestinal M cells of Peyer’s patches. M cells can transport the virus into the intestinal lymphoid tissue where primary replication occurs. Then the infection proceeds with the standard viraemic phase. The bloodstream is accessible through the regional lymph nodes and thoracic duct, and the blood transports the virus to places of secondary replication before the virus reaches the central nervous system as the target organ [94].

The first study showing that the gastrointestinal tract is the gate for the virus was based on the peroral infection of mice was conducted in 1953 [112]. The authors reported that TBEV killed three out of six perorally infected mice. These results were further verified on experiments involving two *Macacus rhesus* monkeys [113]. After peroral inoculation, one monkey showed symptoms of biphasic milk fever. Both monkeys had detectable viraemia and produced specific antibodies. 

For a better understanding of TBEV transmission via the alimentary route, Yu et al., [114] studied Caco-2 cells as an in vitro model to display the interactions between TBEV and human intestinal epithelial cells. Three TBEV strains were used in their study. Rapid virus spread between cells at 48 h p.i. confirmed that TBEV replication is efficient in human intestinal Caco-2 monolayers and that this type of cell is susceptible to TBEV infection. Caco-2 cells showed morphological changes including cytoskeleton rearrangements and cytoplasmic vacuolization after TBEV infection. Virus entry was efficiently blocked by inhibitors of cellular endocytosis pathways and showed characteristics of macropinocytosis. Viruses were detected in the basolateral medium, implying a transcytosis pathway [114]. 

## 10. Conclusions

TBEV is the most significant tick-borne virus in Europe. Alimentary infections mediated by the consumption of raw milk products represent an important proportion of overall TBEV infections and have contributed to the overall increase in TBE incidence in several European countries, particularly those with traditional sheep and goat farming practices. Infected ruminants shed infectious virus into their milk without showing any symptoms. Traditional raw dairy products made in TBEV foci therefore represent a considerable risk to human health. Despite the fact that the alimentary route of TBEV infection has been known of since the 1950s, current knowledge on the course of TBEV infection in domestic ruminants, dynamics of virus shedding in milk, and the extent of alimentary TBEV infections is rather limited and often based on early experimental studies. Nevertheless, experimental data have revealed safe virus inactivating procedures in milk, such as pasteurization and boiling. These practices need to be promoted among farmers as well as consumers, especially in recognized TBEV endemic areas, in order to reduce the incidence of alimentary TBEV infections. 

## Figures and Tables

**Table 1:** Number of cases of alimentary infection of TBEV in European countries.

Country	Reported Time Period	No. of Reported Alimentary Cases	Proportion of Alimentary Cases to All *	Reference
Slovakia	1952–1953	76	23.9% ^a^	[39]
	1951–2000	334	11% ^a,b^	[60]
	2009–2016	163	17%	[61]
	2017–2020	94	16.3%	this review
Croatia	2015	7	27% ^c^	[63]
	2019	6	46% ^c^	[64]
Hungary	2007	31	45% ^e^	[65]
	2011	11	-	[58]
	1953–2011	111	-	[66]
	1992–2011	100	4.4%	[66,89]
Czech republic	1999	21	4.2% ^h^	[67]
	1960–2007	11 ^d^	2.7% ^d^	[68]
	1997–2008	64	0.9%	[69]
Slovenia	2012	3	1.8% ^c^	[59]
Austria	2008	6	6.9% ^f^	[57]
	2020	4	1.9%	[76]
Poland	1993–2008	48	7% ^g^	[78]
	2017	4	2% ^c^	[80]
Estonia	1950–2004	16	-	[82]
	2005	27	16.5% ^f^	[81]
Germany	2016	2	0.6% ^c^	[83]
	2017	13	2.7%	[85]
Romania	1999–2006	17	46%	[87]

* If the proportion was not reported directly in the quoted reference, it has been calculated from the number of reported alimentary cases and all cases reported by the European Centre for Diseases Prevention and Control (ECDC) for the given country and period or in references listed below: ^a^ Grešíková [84]; ^b^ Labuda et al. [60]; ^c^ ECDC (https://www.ecdc.europa.eu/en/search?diseases%5B%5D=191 (accessed on 23 December 2021)); ^d^ only pediatric cases; ^e^ Zöldi et al. [89]; ^f^ Amicizia et al. [90]; ^g^ Czupryna [91]; ^h^ The National Institute of Public Health of the Czech Republic (www.szu.cz (accessed on 23 December 2021)).
